# Luteolin is a novel p90 ribosomal S6 kinase (RSK) inhibitor that suppresses Notch4 signaling by blocking the activation of Y-box binding protein-1 (YB-1)

**DOI:** 10.18632/oncotarget.834

**Published:** 2013-02-27

**Authors:** Kristen M. Reipas, Jennifer H. Law, Nicole Couto, Sumaiya Islam, Yvonne Li, Huifang Li, Artem Cherkasov, Karen Jung, Amarpal S. Cheema, Steven J.M. Jones, John A. Hassell, Sandra E. Dunn

**Affiliations:** ^1^ Laboratory for Oncogenomic Research, Child and Family Research Institute, University of British Columbia, Vancouver, British Columbia, Canada; ^2^ Genome Science Centre, BC Cancer Agency, Vancouver, British Columbia, Canada.; ^3^ Vancouver Prostate Centre, University of British Columbia, Vancouver, British Columbia, Canada.; ^4^ Laboratory for Experimental Oncology, University of Alberta, Edmonton, Alberta.; ^5^ Center for Functional Genomics, McMaster University, Ontario, Canada.

**Keywords:** Triple-negative breast cancer, p90 ribosomal S6 kinase, Y-box binding protein-1, tumor-initiating cells, drug repositioning

## Abstract

Triple-negative breast cancers (TNBC) are notoriously difficult to treat because they lack hormone receptors and have limited targeted therapies. Recently, we demonstrated that p90 ribosomal S6 kinase (RSK) is essential for TNBC growth and survival indicating it as a target for therapeutic development. RSK phosphorylates Y-box binding protein-1 (YB-1), an oncogenic transcription/translation factor, highly expressed in TNBC (~70% of cases) and associated with poor prognosis, drug resistance and tumor initiation. YB-1 regulates the tumor-initiating cell markers, CD44 and CD49f however its role in Notch signaling has not been explored. We sought to identify novel chemical entities with RSK inhibitory activity. The Prestwick Chemical Library of 1120 off-patent drugs was screened for RSK inhibitors using both *in vitro* kinase assays and molecular docking. The lead candidate, luteolin, inhibited RSK1 and RSK2 kinase activity and suppressed growth in TNBC, including TIC-enriched populations. Combining luteolin with paclitaxel increased cell death and unlike chemotherapy alone, did not enrich for CD44^+^ cells. Luteolin’s efficacy against drug-resistant cells was further indicated in the primary x43 cell line, where it suppressed monolayer growth and mammosphere formation. We next endeavored to understand how the inhibition of RSK/YB-1 signaling by luteolin elicited an effect on TIC-enriched populations. ChIP-on-ChIP experiments in SUM149 cells revealed a 12-fold enrichment of YB-1 binding to the Notch4 promoter. We chose to pursue this because there are several reports indicating that Notch4 maintains cells in an undifferentiated, TIC state. Herein we report that silencing YB-1 with siRNA decreased Notch4 mRNA. Conversely, transient expression of Flag:YB-1^WT^ or the constitutively active mutant Flag:YB-1^D102^ increased Notch4 mRNA. The levels of Notch4 transcript and the abundance of the Notch4 intracellular domain (N4ICD) correlated with activation of P-RSK^S221/7^ and P-YB-1^S102^ in a panel of TNBC cell lines. Silencing YB-1 or RSK reduced Notch4 mRNA and this corresponded with loss of N4ICD. Likewise, the RSK inhibitors, luteolin and BI-D1870, suppressed P-YB-1^ S102^ and thereby reduced Notch4. In conclusion, inhibiting the RSK/YB-1 pathway with luteolin is a novel approach to blocking Notch4 signaling and as such provides a means of inhibiting TICs.

## INTRODUCTION

Therapeutic intervention relies on conventional chemotherapeutics for patients with triple-negative breast cancer (TNBC). Since this subtype does not express estrogen receptor (ER), progesterone receptor (PR) or Her-2 patients are ineligible for targeted agents to these molecules such as tamoxifen or trastuzumab. Compared to other subtypes, TNBC has an aggressive clinical course and women with this subtype are faced with the highest recurrence and death rates within the first five years after diagnosis, underscoring the imperative need for new treatments [1-4].

The p90 ribosomal S6 kinases (RSK), particularly RSK1 and RSK2, are associated with breast cancer growth. This family of serine/threonine kinases is part of the MAPK pathway and is responsible for activating a wide range of substrates involved in cell proliferation, motility and survival [5, 6]. Moreover, RSK signaling deregulation may play a role in pre-neoplastic progression to neoplastic disease [7]. RSK1 is primarily known for its role in promoting cancer cell invasion and metastasis [8, 9]. Importantly, RSK2 has recently been identified as a lead molecular target for TNBC [10, 11]. In an unbiased, genome-wide screen for breast cancer subtype-specific inhibitors, RSK2 was one of only three molecules found to be important for sustaining the growth of TNBC [11]. Building on this, we demonstrated that suppressing RSK2 inhibited growth of TNBC cell lines and delayed tumor initiation in mice, providing the first proof-of-concept for RSK2 inhibitors in TNBC [10]. As such, RSK is positioned as a molecular target that could individualize therapy for patients with this breast cancer subtype. However, currently there are no clinically available RSK inhibitors yet a few small molecules have been identified through screening efforts in the past five years [12-15]. Considering the poor prognosis for patients with TNBC, this new information indicating that RSK2 inhibitors could improve treatment of this disease makes a focus in this area timely.

RSK is the predominant kinase that phosphorylates Y-box binding protein-1 (YB-1) at its S102 site [5]. YB-1 is an oncogenic transcription/translation factor that promotes breast cancer growth and drug resistance. Upon phosphorylation at S102, P-YB-1^S102^ translocates to the nucleus and promotes the induction of growth factors such as EGFR, Her-2, and the MET receptor as well as the tumor-initiating-cell (TIC)-associated genes CD44 and CD49f [1]. Indeed, YB-1 may be a signature feature of aggressive forms of breast cancer. We have determined that YB-1 is associated with relapse and poor survival in all breast cancer subtypes, expressed in 60-70% of the most aggressive subtypes (TNBC and Her-2) and is a stronger prognostic marker for breast cancer than those currently used in the clinic [1, 16]. Since YB-1 and P-YB-1^S102^ expression are tightly associated with cancer recurrence we explored the idea this is because YB-1 regulates TIC survival. TICs are hypothesized to be at the root of cancer recurrence as they are resistant to chemotherapy and radiation [17-21]. TICs, by definition, have an increased capacity to initiate tumor formation when transplanted into immunocompromised mice [22]. They make up a subset of the entire tumor ranging from 10%-60% and can be enriched through flow cytometry sorting for cells with CD44^+^/CD24^−^/ESA^+^/CD49f^+^ surface marker phenotype and also through non-adherent mammosphere culture conditions [22-25]. TIC expression correlates with high-grade tumors, is associated with distant metastases and TICs have been detected in circulating tumor cells from women with breast cancer [26]. Further, the CD44-associated gene signature is predictive of poor survival [27]. To support the role of YB-1 in regulating a TIC phenotype, we previously determined that YB-1 binds to the promoters of CD44 and CD49f and induces their expression [17]. Consequently, there is an enhancement of self-renewal and mammosphere growth, as well as an increase in drug resistance in TNBC cells [17]. Conversely, silencing YB-1 decreases CD44 expression and sensitizes cells to chemotherapeutics such as paclitaxel [17]. Collectively, these data point towards YB-1 as a promising molecular target for the treatment of aggressive forms of breast cancer.

TICs exploit many of the same canonical stem cell signaling networks that regulate normal tissue-specific stem cells. In the mammary gland, the Notch signaling pathway plays an important role in development and cell fate determination [28]. The Notch4 isoform in particular has been implicated in mammary stem cells. Notch4 mRNA levels are highest in undifferentiated, bipotent human mammary progenitor cells and decrease upon differentiation [29]. Aberrant expression of the active intracellular domain of Notch4 (N4ICD) prevents differentiation and ultimately induces mammary carcinomas in mice [30]. In breast cancer cell lines and patient samples, CD44^+^/CD24^−^/ESA^+^-sorted TICs express higher levels of activated N4ICD than their non-TIC counterparts [24]. Conversely, expression of Notch1 intracellular domain (N1ICD) is lowest in TICs indicating differential activation of Notch isoforms between TIC and non-TIC populations. Blocking Notch4 specifically using RNA interference reduces the number of CD44^+^/CD24^−^/ESA^+^ cells, suppresses mammosphere formation and completely inhibits tumor initiation whereas inhibiting Notch 1 has only a modest effect [24, 31]. Interestingly, YB-1 binds to the promoters of several stem-cell-associated genes including Notch4 yet, YB-1’s role in regulating TICs through Notch4 signaling remains to be explored [32].

However, with no small molecules or drugs to directly inhibit YB-1, we instead sought to block RSK kinase activity and thereby prevent phosphorylation of YB-1. We have demonstrated that inhibiting YB-1 using this approach is effective at eliminating TICs [10]. Further, since translating the use of RSK/YB-1 inhibitors into the clinic would be costly and time consuming, we questioned whether existing drugs had RSK-inhibitory activity. As the underlying mechanisms driving carcinogenesis become better understood, repositioning currently approved drugs for the treatment of cancer has become an area of interest [33-35]. One of the best examples is the identification of salinomycin, which was derived from a screen of 16,000 off-patent compounds in a search to find new opportunities to inhibit breast TICs [36]. In another example of drug repositioning, the anti-diabetic drug metformin was shown to inhibit the growth of breast TICs [37, 38] and prevent relapse in xenograft models of prostate and lung cancer [39]. Disulfiram, a drug used to manage alcoholism, has also been described as being able to kill CD44^+^ cells in models of breast cancer [40]. We therefore hypothesized that there may currently be existing compounds that would inhibit the RSK/YB-1 pathway. To this end, we screened the Prestwick Chemical Library of 1120 off-patent drugs in RSK kinase assays and molecular docking. Two major advantages of this drug collection are that 85% of these chemicals are FDA approved and the safety, bioavailability and dosing schedules are established, making the transition from initial screening to drug application more efficient. Herein, we identified luteolin as having novel RSK inhibitory activity with the ability to block YB-1/Notch4 signaling and suppress growth in TIC-enriched populations.

## RESULTS

### Screening of Prestwick Chemical Library identified novel RSK inhibitors

We screened the Prestwick Library consisting of 1120 chemicals at 10 μM in an *in vitro* RSK1 kinase assay against the YB-1 peptide containing the S102 site. The YB-1 peptide was selected because it was previously characterized for binding to RSK1 using *in vitro* kinase assays [5] and through molecular docking [41]. Thirty-two compounds were identified that inhibited RSK1 kinase activity >20% at 10 μM (Supplemental Table 1). When compared to the short list from the *in silico* screen (including the 25 strongest predicted binders), 3 compounds were indicated in both screens: kaempferol, luteolin and apigenin (Table 1 and Supplemental Table 1). The molecular docking screen theoretically identify compounds that would inhibit RSK kinase activity using Glide and ICM docking software which consistently rank the highest in terms of docking scoring and accuracy [42, 43]. A crystal structure of RSK1 bound to ATP in the N-terminal kinase domain (2Z7Q.pdb) was used to predict that kaempferol, apigenin, luteolin bind to the kinase in its active conformation. Importantly, using this RSK1/ATP structure, kaempferol, apigenin and luteolin were predicted to bind to RSK1 at Leu144 and Asp142, both of which are the major sites for ATP binding in the NTKD (Table 1) [44]. Apigenin and luteolin were also predicted to bind to Gln70. Relative to all of the drugs in the Prestwick Library, apigenin and luteolin ranked in the top ~1%, scoring higher than kaempferol (Table 1). The docking results were independently confirmed against two additional RSK1 structures in active conformations, RSK1 co-crystallized to staurosporine, and purvalanol A (Supplemental Table 2). Taken together, we used biochemical screens and computational docking to short-list three agents that inhibited RSK at the NTKD. Kaempferol, apigenin and luteolin are all flavonoid analogues with remarkably similar structure, sharing a common backbone and differing only in hydroxy group location (Table 1). Kaempferol has known RSK inhibitory activity [12] and therefore it served as an unbiased internal control.

**Table 1:** Molecular docking supports ability of drugs to block RSK1 activity Binding models for the lead compounds in relationship to the RSK1 NTKD. The RSK1 structure was obtained by co-crystallization with ATP. The major binding sites for ATP are Leu 144 and Asp 142. Notably kaempferol, apigenin and luteolin all bind to these sites. Luteolin and apigenin also bind to Gln 70 and Thr 204 while kaempferol binds to Asp 205. The binding mode and theoretical H-bonds are shown as well as the Glidescore and rank of the lead compounds in the Prestwick Library.

Compound	Docking using RSK1 N-terminal kinase domain conformation co-crystallized with ATP (2Z7Q.pdb)			
	Binding Mode	sp	rank^1^	%^2^
**Apigenin**	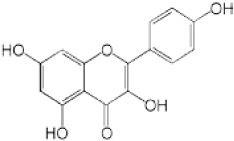	−7.99	6	0.54
**Luteolin**	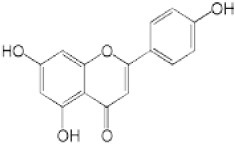	−7.63	10	1.34
**Kaempferol**	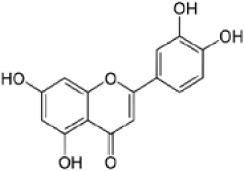	−7.54	25	2.23

^1^ the ranking of the compound among the 1120 Prestwick Chemical Library

^2^ the percentage of the compound among the 1120 Prestwick Chemical Library

Note: that the agents identified ranked in the top 1% of potential binders

Following the RSK1 screen, a broad dose-response study (0.001-100 μM) was conducted against RSK2 using the YB-1 peptide as a substrate in cell-free assays (Table 2). Each of the agents inhibited its activity with similar IC_50_ values ranging from 1.71-4.77 μM. BI-D1870 was included as a positive control as it is known to inhibit RSK1 and RSK2 [45] (Table 2). To further validate these data, the same range of concentrations was assessed using a secondary RSK substrate, the S6K peptide, in both the RSK1 and RSK2 kinase assays (Supplemental Table 3A-B). Similarly, all three compounds inhibited the kinase activity of both isoforms in the low micromolar range. While the flavonoids were less potent than BI-D1870, they are favored because they are commercially available as dietary supplements and their safety/toxicity profiles are established [46]. Conversely, BI-D1870, while it is a potent RSK inhibitor, has never been tested in animals or humans to our knowledge.

**Table 2:** Kaempferol, apigenin and luteolin block RSK2 kinase activity The lead candidates (0.001 μM-100 μM) were analyzed in an *in vitro* RSK2 kinase assay against the YB-1 peptide as the substrate. The IC_50_ for each was determined. Chemical structures for these candidates are shown. BI-D1870 was used as a positive control.

		Inhibition of RSK2 (%) against the YB-1 peptide as the substrate						
Compound	Structure	0.001 μM	0.01 μM	0.1 μM	1.0 μM	10 μM	100 μM	IC_50_ (μM)
Kaempferol	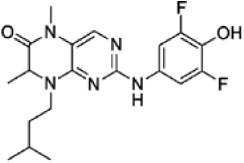	1	9	16	42	77	94	1.71
Apigenin	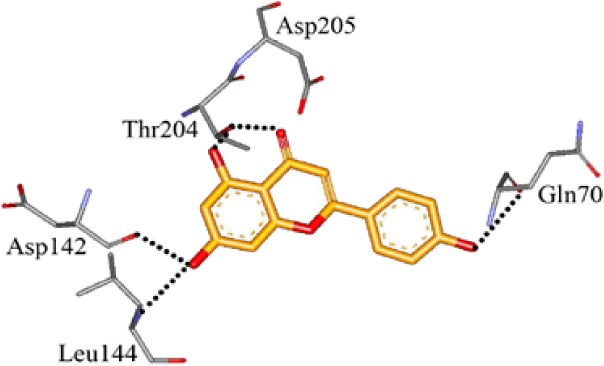	−2	−2	11	32	62	79	4.77
Luteolin	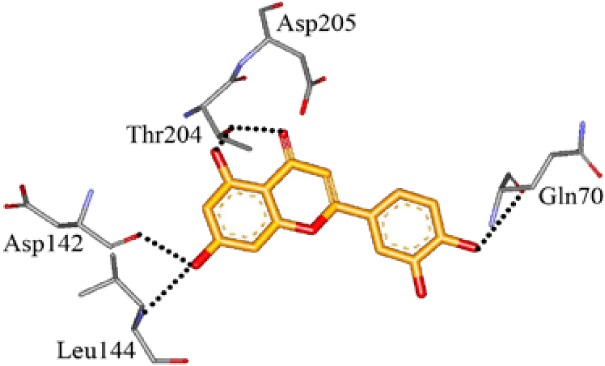	2	5	8	26	67	83	4.42
BI-D1870	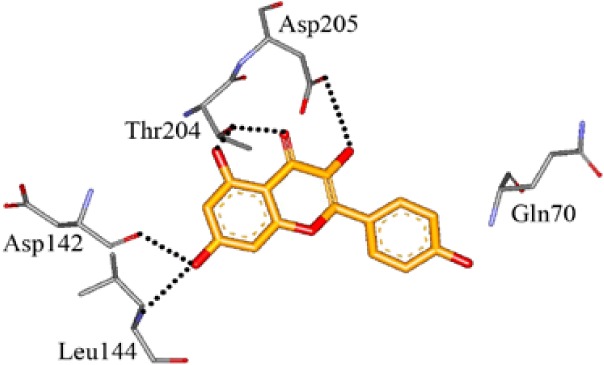	14	49	76	93	100	100	0.016

### Luteolin prevents activation of YB-1 and subsequently inhibits proliferation, anchorage-independent growth and mammosphere formation in TNBC

**Figure 1:**
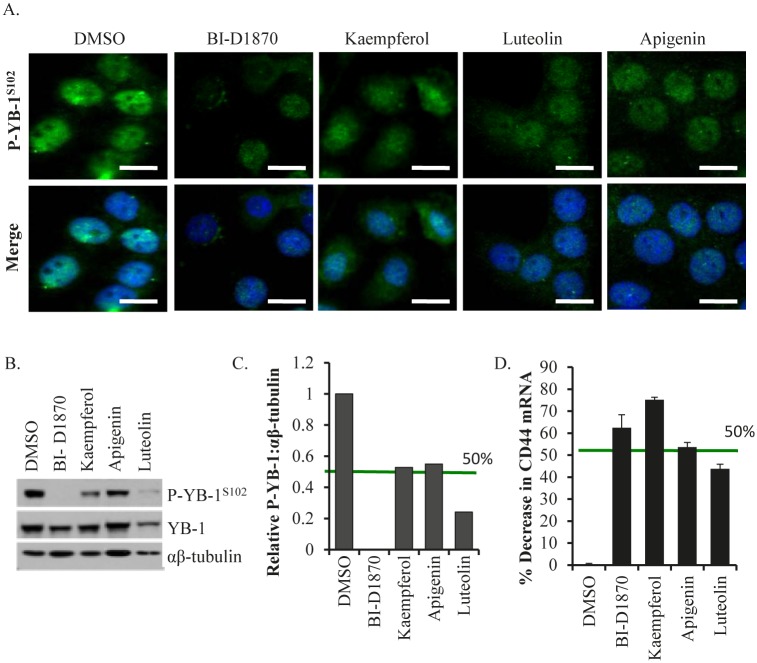
Lead compounds block activation of YB-1 A) Drug treatments (10 μM/24 h) reduced nuclear P-YB-1^S102^ based on changes in immunofluorescence. Scale bar is 15 μm. B) Following drug treatment (10 μM/24 h), cells lysates were analysed by immunoblotting for P-YB-1^S102^. BI-D1870 was used as a positive control. C) Immunoblots were scanned and the P-YB-1^S102^ band intensities were normalized to αβ-tubulin. D) The YB-1 downstream target and TIC marker, CD44, transcript level were reduced with drug treatments (10 μM/48 h).

In the secondary screens, we investigated the lead compounds for their ability to block P-YB-1^S102^ in the TNBC cell line SUM149. In the absence of the inhibitors, activated YB-1 was present in the nucleus (Figure 1A). When cells were treated with the lead compounds, P-YB-1^S102^ immunofluorescence was diminished compared to the DMSO control (Figure 1A). Additional images are provided to illustrate that this was a general effect of the inhibitors (Supplemental Figure 1). Further, immunoblotting confirmed that P-YB-1^S102^ was decreased with drug treatment (Figure 1B). The blots were scanned, normalized to αβ-tubulin and quantified. Luteolin inhibited P-YB-1^S102^ by ~80% at 24 h (Figure 1C). We next evaluated the YB-1 downstream target, CD44, by qRT-PCR. This target is particularly important, as it has been shown to be associated with a TIC signature ([1]; plus references therein). All of the lead compounds decreased CD44 transcript levels (Figure 1D). These results paralleled those of an established RSK inhibitor, BI-D1870.

**Figure 2:**
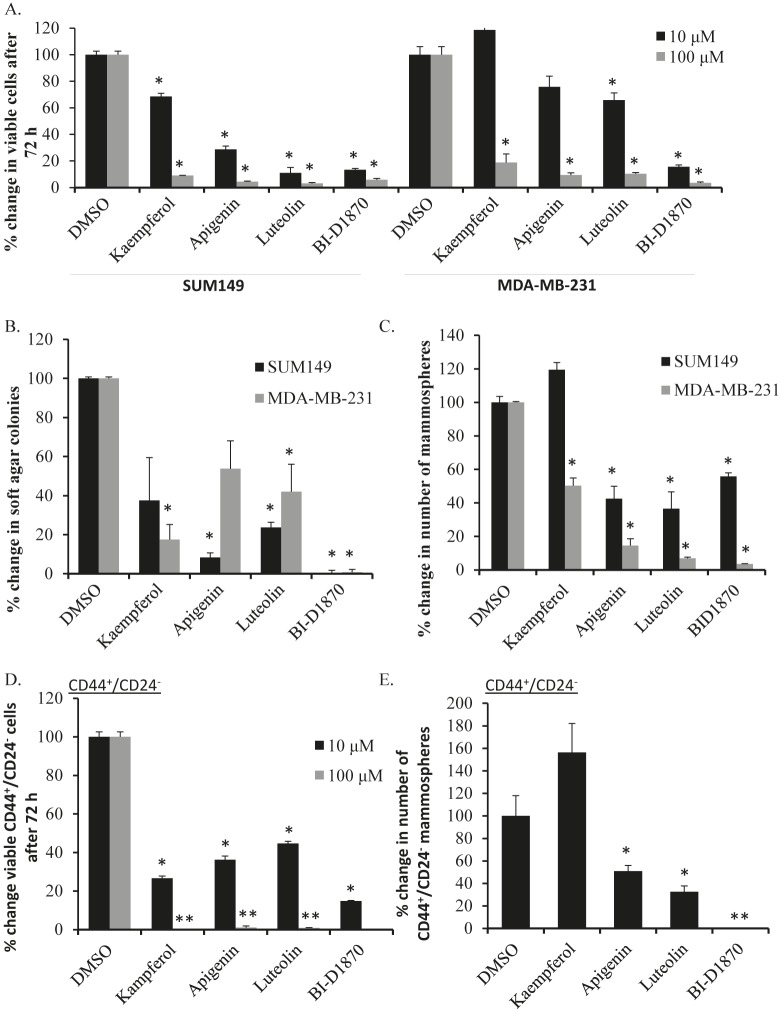
Lead compounds inhibit growth in TNBC models and CD44^+^/CD24^−^ cells A) SUM149 and MDA-MB-231 cells were treated in triplicate with 10 μM or 100 μM of drug. After 72 h, the number of cells was counted and normalized to the DMSO control. B) The soft agar assay was established with drugs (10 μM) added to the top layer at the time of seeding. Colonies were counted at 28 d. C) Lead compounds (10 μM) were assayed in mammosphere conditions, which enrich for tumor-initiating cells. Mammospheres formed were counted after 7 d and expressed as a percent relative to DMSO control. D) CD44^+^/CD24^−^-sorted SUM149 cells were treated with lead compounds (10 μM or 100 μM) in monolayer and E) mammosphere conditions as described above. BI-D1870 (10 μM), a known RSK inhibitor, was used as a positive control in all assays. Students T test was used to determine statistical significance (* *P*<0.05, ** *P*<0.005)

We recently published that inhibiting RSK and thereby blocking the activation of YB-1, leads to decreased growth in TNBC [5, 10]. We therefore assessed lead compounds for growth effects in models of TNBC (SUM149 and MDA-MB-231 cells). In these cells, monolayer growth was significantly decreased with 10 μM luteolin (Figure 2A). At 100 μM, kaempferol and apigenin were added to this list. No growth effect was observed in normal immortalized mammary epithelial cells (184hterts) at 10 μM (Supplementary Figure 2). We then tested kaempferol, apigenin and luteolin in a soft agar assay at 10 μM. These three compounds all showed significant inhibition of colony formation under anchorage-independent conditions in at least one cell line with luteolin significantly inhibiting colony formation in both (Figure 2B). We next assessed the compounds in mammosphere assays. Anoikis-resistant cells have increased tumor-initiating capacity *in vivo* validating this culture technique as a method of enriching for TICs [24]. SUM149 mammosphere formation was significantly inhibited in the presence of 10 μM, apigenin or luteolin (Figure 2C). Kaempferol reduced the number of mammosphere formed by about 50% in the MDA-MB-231 cells but had limited effect on SUM149 mammospheres. To address this seemingly discordant result, we questioned whether kaempferol would inhibit mammosphere formation upon serial passaging, which it did (~50%) by the tertiary passage when compared to the number of DMSO-treated primary mammospheres (Supplemental Figure 3A). Alternatively, we tested whether daily dosing would improve kaempferol’s ability to inhibit mammosphere formation, as this drug may be less stable in this cell culture assay and found that this protocol also inhibited mammosphere formation (Supplemental Figure 3B). We next asked whether these compounds could inhibit growth of mammospheres once they were already established. Apigenin and luteolin showed a marked reduction in the number of mammospheres in SUM149 cells and MDA-MB-231 cells (Supplemental Figure 3C-D). Kaempferol however, had little effect on mammospheres once established in both cell lines supporting the idea that although they share similar backbones, differences in hydroxy groups between the compounds alters the structure-activity relationship regarding RSK inhibitory potential (Table 2, Supplemental Figure 3C-D). Likewise, the positive control (BI-D1870) inhibited mammosphere formation and colony growth in soft agar (Figures 2B-C and Supplemental Figure 3C-D).

### Luteolin blocks growth of TIC-enriched populations and primary relapsed TNBC cells

SUM149 cells were sorted for CD44^+^/CD24^−^ TIC-enriched fractions as described [22, 23] and subsequently treated with the lead drugs and evaluated by immunofluorescence for P-YB-1^S102^ (Supplemental Figure 4). Consistent with un-sorted cells, P-YB-1^S102^ was predominantly expressed in the nucleus of the DMSO treated cells, yet each of the drugs causes marked elimination of it from the nuclear compartment (for additional images see Supplemental Figure 5). Further, the lead agents significantly inhibited monolayer growth of CD44^+^/CD24^−^ cells after 72 h (Figure 2D). The number of mammospheres formed by CD44^+^/CD24^−^ cells was reduced by treatment with luteolin and apigenin but not kaempferol (Figure 2E). Since differentiation of CD44^+^/CD24^−^ sorted populations occurs rapidly, cells were seeded into growth assays immediately after sorting. The control agent BI-D1870 also suppressed P-YB-1^S102^ and growth of CD44^+^/CD24^−^ cells in monolayer and in mammosphere cultures (Figure 2D-E, Supplemental Figure 4-5).

**Figure 3:**
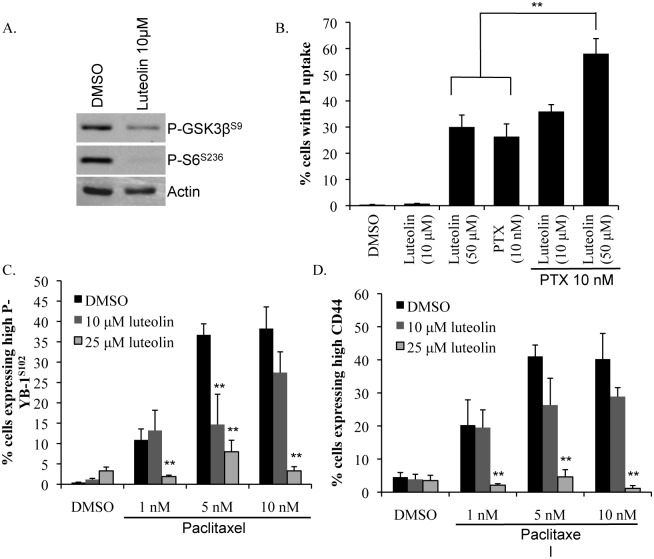
Luteolin prevents enrichment for CD44+ cells by paclitaxel A) Luteolin (10 μM) inhibited phosphorylation of RSK downstream targets GSK3β and S6 at 24 h as demonstrated by immunobloting. B) Combining luteolin (10 μM or 50 μM) with paclitaxel (PTX) (10 nM) increased PI uptake. SUM149 cells were treated for 72 h and PI uptake was assessed using Cellomics ArrayScan VTI. C) Adding luteolin (10 μM or 25 μM) to paclitaxel (PTX) (1-10 nM) treatment prevented the induction of P-YB-1^S102^ and D) CD44 by paclitaxel in SUM149 cells. Cells were treated for 72 h (**P<0.005).

When the results from all levels of screening were taken collectively, luteolin was identified as the lead candidate as it: 1) ranked in the top ~1% out of 1120 chemicals in the *in silico* RSK1 docking, 2) interacted with critical ATP binding residues in the RSK1 NTKD, 3) demonstrated ~80% knockdown of P-YB-1^S102^ protein at 24 h, 4) suppressed growth of both TNBC cell lines in monolayer, soft agar and mammosphere culture conditions and 5) inhibited growth of CD44^+^/CD24^−^ cells in monolayer and mammospheres. As such, luteolin underwent further evaluation. Two additional RSK substrates, P-GSK3β^S9^ and P-S6^S236^, were also confirmed reduced after treatment with luteolin at 24 h (Figure 3A). Additionally, luteolin’s inhibition of P-YB-1^S102^ was dose-dependant (Supplemental Figure 6). Since luteolin has been shown to interact with proteins across several biological pathways, we compared the predicted binding of luteolin and RSK to other potential targets [47, 48]. When luteolin was docked against 252 known drug targets, RSK ranked highest among the list (Supplemental Table 4). Although luteolin was predicted to bind to other targets in addition to RSK, some of these “off-target” proteins may have added benefit for cancer therapy as they have also been implicated in cancer survival. For example, KIT was identified as a putative luteolin target. KIT is a cytokine cell-surface receptor that binds to stem cell factor and has been indicated as an emerging therapeutic target for breast cancer therefore may itself be effective at treating this disease [1].

We next wanted to test the effect of combining luteolin with a front-line chemotherapeutic, paclitaxel. An undesirable effect of paclitaxel is that it activates RSK/YB-1 signaling and subsequently up-regulates CD44 expression [17]. Moreover, clinical observation and studies in cell lines have demonstrated that taxanes enrich for CD44^+^/CD24^−^ cells indicating ineffective targeting of this population [18, 23]. Conversely, our data suggest that luteolin actually suppresses growth of CD44^+^/CD24^−^ cells. Luteolin has also been reported to be a chemosensitizing agent [49, 50]. We therefore hypothesized that the addition of the RSK inhibitor luteolin would not only increase the sensitivity of cells to paclitaxel but also eliminate the CD44^+^ cells. Indeed, we found that the combination of luteolin (50 μM) with paclitaxel (10 nM) significantly increased cell death compared to either drug alone as indicated by PI uptake (Figure 3B). Moreover, including luteolin in the regimen prevented activation of P-YB-1^S102^ and enrichment of CD44^+^ cells by paclitaxel (Figure 3C-D). These data suggest that the addition of a RSK inhibitor such as luteolin to paclitaxel is an effective strategy to improve cell death and reduce the residual CD44^+^ cell burden.

**Figure 4:**
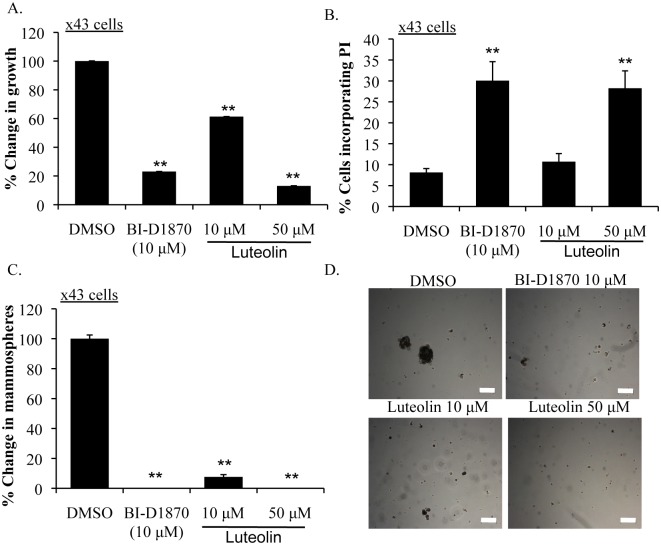
Luteolin inhibits growth and induces apoptosis in primary human TNBC A) Treating the primary relapsed human TNBC cell line x43 with luteolin (10 μM or 50 μM) suppressed growth by ~90% at 72 h. BI-D1870 (10 μM) was used as a positive control (**P<0.005). B) Treating x43 cells with either luteolin (10 μM or 50 μM) or BI-D1870 (10 μM) for 72 h increased PI uptake (**P<0.005). C) Luteolin (10 μM or 50 μM) and BI-D1870 (10 μM) inhibited mammosphere formation counted at 7 d (**P<0.005). D) Representative pictures of mammospheres. Scale bar = 200 μM.

Extending these findings to primary TNBC, we tested the efficacy of luteolin in the x43 cell line, derived from a patient who suffered relapse thus suggesting this may be an aggressive case. Subtype classification was confirmed by NanoString (Supplemental Figure 7). The x43 cells had low levels of ER, PR and Her-2 mRNA when compared to Her-2 over-expressing (HR6) cells and had marker expression that was similar to two TNBC cell lines (HCC1143 and MDA-MB-231). Moreover, the x43 cells represent a basal-like breast cancer as they express EGFR, Keratin 5 and Keratin 6A (Supplemental Figure 7). Treating x43 cells with luteolin suppressed growth by up to ~90% at 50 μM (Figure 4A). A similar effect was seen with positive control BI-D1870. Growth inhibition corresponded to a reduction in P-YB-1^S102^ beginning at 24 h at 10 μM (Supplemental Figure 8). Luteolin induced cell death at these concentrations as indicated by PI uptake (Figure 4B). Moreover, x43 cells were exquisitely sensitive to RSK inhibition in non-adherent conditions, as luteolin completely blocked the ability to form mammospheres at 10 μM (Figure 4C-D).

### YB-1 regulates Notch4 expression and can be abrogated through RSK inhibition using luteolin

**Figure 5:**
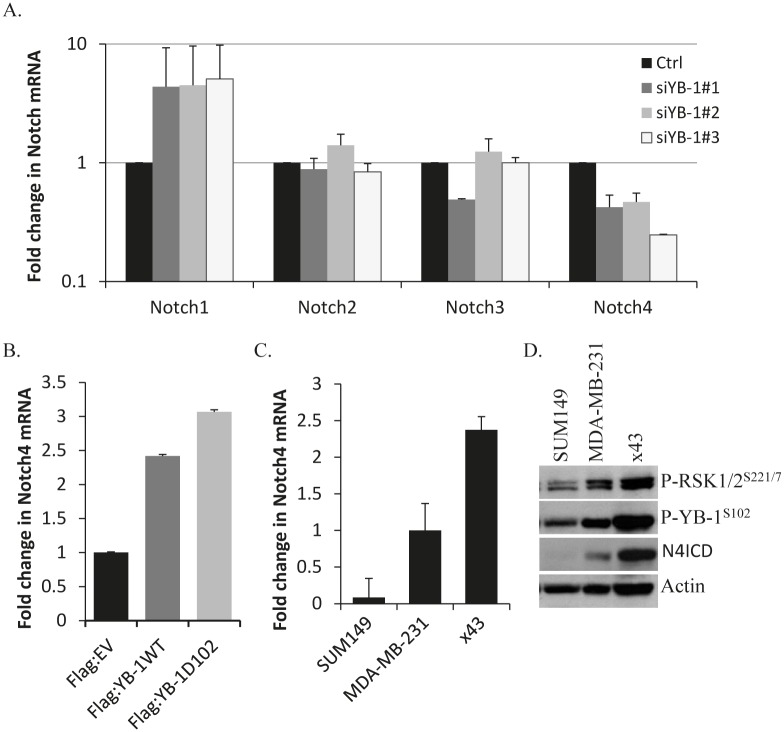
Notch4 transcript levels are reduced by blocking YB-1 signaling and correlate with P-YB-1^S102^ and P-RSK^S221/7^ A) Inhibiting YB-1 using siRNA reduced Notch4 mRNA levels. SUM149 cells were transfected with three distinct siRNA’s against YB-1 and all four Notch isoforms were assessed using quantitative real-time PCR 96 h after transfection. B) Transient transfection of SUM149 cells with either Flag:YB-1^WT^ or Flag:YB-1^D102^ plasmids for 96 h increased Notch4 levels compared to control empty vector. C) Expression of Notch4 mRNA and D) active intracellular domain (N4ICD) correlates with P-YB-1^S102^ and P-RSK^S221/7^ in a panel of TNBC cell lines (SUM149, MDA-MB-231, primary x43).

To begin to understand the mechanism by which RSK inhibitors elicited an effect on TIC-enriched populations we identified putative YB-1 target genes using ChIP-on-ChIP assays. These studies revealed that YB-1 binds to the promoters of several TIC-associated genes. Most notably, we found a 12-fold enrichment of YB-1 binding to the Notch4 promoter [32]. Reports from others demonstrate that Notch4 signaling is elevated in CD44^+^/CD24^−^ cells and that inhibiting this pathway reduces mammosphere formation and prevents tumor initiation *in vivo* identifying Notch4 as a critical regulator of breast cancer TICs [24, 31]. The prominent role of Notch4 in TICs hinted that luteolin’s efficacy against CD44^+^/CD24^−^ cells may be through inhibition of YB-1 and thereby suppression of Notch4. To confirm our ChIP-on-ChIP results, we tested the effect of YB-1 knockdown on all of the Notch isoforms (Notch1-Notch4). Knockdown of YB-1 using three different siRNAs increased Notch1 mRNA and decreased Notch4 mRNA with no effect on the Notch2 or Notch3 isoforms in SUM149 cells (Figure 5A). Conversely, over-expression of either wild-type YB-1 (Flag:YB-1^WT^) or a constitutively active mutant YB-1 (Flag:YB-1^D102^) in SUM149 cells increased levels of Notch4 mRNA (Figure 5B, Supplemental Figure 9 for control blot). Interestingly, when comparing a panel of TNBC cell lines (SUM149, MDA-MB-231 and x43) the level of Notch4 mRNA and cleaved, activated, intracellular domain (N4ICD) correlated with the levels of P-YB-1^S102^ and P-RSK^S221/7^ (Figure 5C-D).

**Figure 6:**
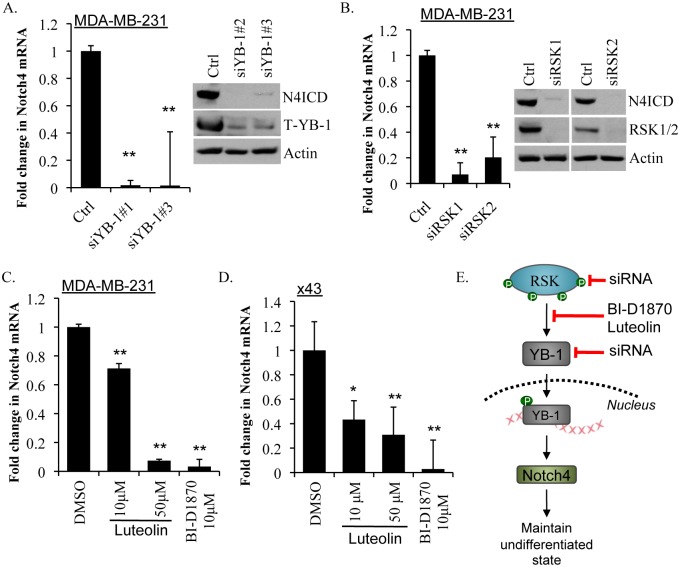
Inhibiting YB-1, RSK1 or RSK2 or blocking activation of YB-1 with RSK inhibitors repressed Notch4 mRNA and N4ICD A) YB-1 knockdown using siRNA reduced Notch4 transcript levels in MDA-MB-231 cells at 48 h and decreased N4ICD protein levels by 72 h. B) Knockdown of RSK1 or RSK2 reduced Notch4 transcript levels in MDA-MB-231 cells at 48 h and decreased N4ICD protein levels by 72 h. C) RSK inhibitors luteolin (10 μM or 50 μM) and BI-D1870 (10 μM) also decreased expression of Notch4 transcript in MDA-MB-231 cells and D) primary x43 cells at 48 h. E) A schematic diagram of luteolin and BI-D1870 inhibiting the RSK/YB-1/Notch4 pathway.

Building on the idea that the RSK/YB-1 pathway regulates Notch4 signaling we investigated the levels of Notch4 after YB-1 knockdown in the MDA-MB-231 cells (since this cell line expresses more Notch4 than the SUM149). Reducing YB-1 using siRNA decreased Notch4 mRNA and correspondingly decreased N4ICD levels (Figure 6A). Similarly, knockdown of either RSK1 or RSK2 also reduced Notch4 mRNA and N4ICD (Figure 6B). This effect was further demonstrated in a second cell line (x43) where knockdown of either RSK1, RSK2 or YB-1 suppressed Notch4 transcript (Supplemental Figure 10). The RSK inhibitors BI-D1870 and luteolin paralleled these results and significantly reduced Notch4 mRNA in both the MDA-MB-231 and the x43 cell lines (Figure 6C-D). Thus, we conclude that RSK inhibition decreases Notch4 signaling by suppressing P-YB-1^S102^ (Figure 6F). x43

## DISCUSSION

Herein, we identified the off-patent compound luteolin, has the novel ability to block RSK/YB-1/Notch4 signaling and thereby inhibit TNBC growth including TIC-enriched populations. Since RSK has recently been identified as a TNBC-specific target, we focused on screening for compounds that have the ability to block RSK activity. We used a dual approach of high-throughput and virtual screening, as these are complementary methods that can be integrated to improve inhibitor discovery [51]. Notably, both screening techniques identified kaempferol, luteolin and apigenin that inhibited RSK1 and RSK2 at micromolar concentrations. Subsequent experiments identified luteolin as the lead compound as it suppressed growth in TNBC and inhibited RSK in cells. Consequently, it reduced phosphorylation of YB-1 and decreased Notch4 signaling, both of which are key pathways in sustaining TICs.

RSK2 is an emerging therapeutic target for developing treatments for TNBC, for which there are currently no targeted therapies available [11]. Our group identified that RSK2 specifically has the most potent inhibitory effect on growth in TNBC [10]. Furthermore, we propose that RSK inhibitors could have application beyond breast cancer to include other tumors that express high RSK2 such as those that develop in the lung, head and neck, prostate and hematopoietic system [52]. Several other groups have demonstrated that the RSK2 isoform appears to be the most relevant in cancers [53]. In an effort to identify new RSK2 specific inhibitors, Liu *et al* (2011) identified eriodictyol through molecular docking [13]. Interestingly, eriodictyol is a flavonoid that is structurally very similar to, luteolin, apigenin and kaempferol. Similarly, Berghe *et al* (2011) found the flavanone, lavandylyl, to attenuate the ERK/RSK-2 pathway suggesting that there is a structural basis for flavonoids in inhibiting RSK2 [15]. Flavonoids have also been indicated as promising anti-cancer agents in recent years. Additionally, they have shown evidence of having anti-oxidant, anti-bacterial, anti-viral, anti-inflammatory, and anti-cancer beneficial health effects [54]. These naturally occurring compounds are therefore attractive starting points for synthesizing analogues that have higher selectivity and potency. Herein, we identified the flavones luteolin and apigenin with remarkably similar structure had significant growth effects on TNBC. Kaempferol, which is also a flavonoid, served as the basis for making SL0101, an agent that is now utilized as a RSK inhibitor for research purposes [14]. Thus, kaempferol served as an excellent unbiased control in this study. The similarity in structure of all the compounds and the fact that they were identified in both *in vitro* kinase assays and *in silico* docking to an ATP-bound RSK crystal structure is indicative of a structure-activity relationship for these compounds as ATP competitive inhibitors against RSK. Accordingly, we demonstrate that luteolin blocks phosphorylation of a synthetic YB-1 peptide by RSK1 and RSK2 as well as phosphorylation of YB-1 in cancer cells and its nuclear localization. It also suppresses growth in TNBC models. Luteolin has previously been shown to have anti-cancer properties such as suppressing cell survival pathways while promoting apoptosis [47, 48]. This compound is found naturally in many fruits and vegetables and thus is considered safe for consumption [47, 48]. *In vivo* experiments have also demonstrated low toxicity with long-term treatment [47]. Moreover, it is sold commercially as LutiMax, a nutraceutical that has reported benefits not only for cancer but also for other disorders ranging from inflammation to neurologic conditions such as autism. The recommended dosing of Lutimax is 400-600 mg/day with no reported toxicities. The commercial availability of luteolin sold as LutiMax potentially provides a means of translating our research to patients.

Luteolin is documented to alter several biological pathways [47, 48]. To attempt to identify other putative binding proteins, luteolin was docked to a library of 252 known drug targets. RSK scored highest within this library with the strongest predicted binding to luteolin. While we do not disregard that some of luteolin’s anti-cancer effects may be through alternate signaling pathways, we do posit that its activity as a RSK inhibitor could have a particularly significant effect in the context of TNBC given that RSK signaling is critical to the survival of this breast cancer subtype [10]. Moreover, some of the other signaling pathways that luteolin affects could be linked to RSK inhibition. Several studies show that luteolin inhibits NF-κB signaling and sensitizes cells to tumor necrosis factor (TNF)-induced apoptosis [55, 56]. Interestingly, RSK regulates NF-κB signaling through IκB kinase (IKK), which phosphorylates IκB, targeting it for degradation and thereby allowing NF-κβ to translocate to the nucleus and transcribe anti-apoptotic genes [57]. Therefore, inhibition of RSK kinase activity presents one feasible mechanism by which luteolin could inhibit NF-κβ signaling. Furthermore, some of the putative targets we identified for luteolin also play a role in cancer, particularly in TNBC. KIT is co-expressed with EGFR and is associated with *BRCA1*-mutation carriers and in sporadic basal-like breast cancer [58, 59]. Perhaps, by targeting several biological pathways, luteolin could prevent the development of *de novo* resistance in cancer cells that can occur when cells circumvent the requirement for single pathways targeted by highly specific inhibitors.

TICs present a major obstacle in developing effective cancer treatments as many conventional therapies actually enrich for CD44^+^ cells [18, 19, 21]. However, reducing YB-1 expression or preventing its activation via RSK inhibition, are both effective strategies for reducing the TIC burden [10, 17]. As such we investigated whether luteolin could suppress growth in CD44^+^/CD24^−^ cells through inhibition of the RSK/YB-1 pathway. Additionally, since luteolin suppresses cell survival mechanisms and induces apoptosis in cancer cells we speculated that it might work as chemosensitizing agent in conjunction with chemotherapeutics. It has demonstrated this property in other cancers including in gastric cancer when used in combination with cisplatin [49]. Herein, we demonstrate that the addition of luteolin to paclitaxel increased cytotoxicity in TNBC. Importantly, unlike paclitaxel alone the combination of the two compounds did not enrich for CD44^+^ cells. Moreover, luteolin suppresses growth, induces apoptosis and inhibits mammosphere formation in primary human TNBC cells. TICs also play an important role in mediating drug resistance in other breast cancer subtypes. In mice, the addition of luteolin reversed doxorubicin resistance in MCF-7 and 4T1 cells. It also remarkably enhanced the effect of doxorubicin on tumor suppression [50]. In this study, luteolin was actually safer as a single agent and more effective than doxorubicin [50]. We have shown that RSK and YB-1 are up-regulated in trastuzumab-resistant cell lines HR5 and HR6 when compared to their sensitive counterpart BT474 [60]. Expression of an active mutant YB-1^D102^ induced expression of CD44 and conveyed trastuzumab insensitivity to BT474 cells. Conversely, reducing CD44 in HR5 and HR6 cells restored sensitivity to trastuzumab. Thus, combining luteolin with currently used chemotherapeutics may present an effective strategy for eliminating TICs across several breast cancer subtypes.

The Notch family of transmembrane receptors, particularly Notch4, has been implicated in mammary stem/progenitor cell self-renewal and expansion [28]. In both the normal mammary gland and breast cancer, Notch4 signaling maintains an undifferentiated, stem/progenitor-like state [24, 29-31]. Previous experiments revealed that YB-1 binds to the promoter of Notch4 and increases its expression [32]. Herein, we show that inhibiting RSK/YB-1 signaling with siRNA or small molecules reduces Notch4 levels and activation. Harrison *et al* (2010) demonstrate that Notch4 signaling is highest in TICs whereas Notch1 signaling is highest in non-TIC fractions [24]. Interestingly, YB-1 knockdown mimics this pattern of expression and reduces Notch4 mRNA while increasing Notch1 mRNA levels. Constitutive Notch4 signaling promotes an aggressive malignant phenotype in MDA-MB-231 cells increasing vascularization and growth of xenograft models [61]. Conversely, inhibiting Notch4 using antibodies specific to this isoform is more effective at suppressing mammosphere formation than γ-secretase inhibitors which inhibit all Notch isoforms (Notch1-Notch4) indicating that the Notch4 isoform specifically, is important for mammosphere forming ability [31]. Furthermore, the γ-secretase inhibitor, MRK003, was recently shown to inhibit tumor initiation in mice using an ErbB2 model of mammary tumorigenesis and mice treated with MRK003 had durable long-term relapse free survival [62]. Collectively, these data point to an essential role for Notch4 in cancer recurrence through the maintenance of TICs.

We conclude that drug repositioning can be used to identify agents for molecular targets such as RSK. We identify luteolin as having the novel ability to inhibit RSK/YB-1 activation and suppress Notch4 signaling. The discovery of RSK-specific inhibitors that can be fast-tracked into clinic may have significant implications for treating TNBC, where the disease is aggressive and targeted therapies are unavailable. This is an important advance because luteolin inhibits RSK activity and is commercially available as LutiMax. This preclinical study provides the rationale for addressing the potential for luteolin for the treatment of TNBC *in vivo* and in a clinical trial setting.

## MATERIALS AND METHODS

### Initial RSK1 kinase screens and chemicals

For RSK1, the entire Prestwick Chemical Library (1120 chemicals; Canadian Chemical Biology Network at the University of British Columbia) was screened by SignalChem (Richmond, BC) in a kinase assay at 10 μM against a YB-1 peptide, PRKYLRSVG, [41] as previously described [5]. This peptide contains the YB-1 S102 site. Results were compared to a staurosporine control, a broad-spectrum kinase inhibitor that has 100% activity at 10 μM. Compounds with >20% inhibitory activity were considered to be significant RSK1 inhibitors. To confirm RSK1 kinase inhibition, we repeated the kinase assay using a secondary RSK substrate, S6K. Kaempferol, apigenin and luteolin were purchased from Sigma-Aldrich Chemical (Oakville, ON) and were dissolved in DMSO (Sigma) to stock concentrations of 100 mM. BI-D1870, a known RSK inhibitor [45], was synthesized by the Center for Drug Research and Development (Vancouver, BC).

### *In silico* RSK1 screens

*In silico* analysis was performed on lead compounds using the molecular docking program Glide [63, 64]. The docked poses were ranked based on docking score. The Glide docking was performed as follows: For the three crystal structures of N-terminal domain, the cognate ligands were used to define the active sites and generate the grid. Both Glide standard precision (SP) and extra precision (XP) modes were used for the docking, and for each ligand, the highest scored pose was written out. We used three different resolved crystal structures of RSK1, all of which are in the active conformation of the N-terminal domain (2Z7Q.pdb, 2Z7R.pdb, 2Z7S.pdb) co-crystallized with different ligands (ATP, staurosporine, and puravalnol A). Other parameters in Glide were kept at the default setting.

### RSK2 kinase

Kinase profiling services for RSK2 were provided by SignalChem, as previously described [5]. Briefly, the compounds kaempferol, apigenin, luteolin and BI-D1870 were screened in a RSK2 kinase assay at 0.001, 0.01, 0.1, 1.0, 10, and 100 μM against a YB-1 peptide containing the S102 site [41]. Results were compared to a staurosporine control. For each compound, a graph of log concentration (μM) versus % inhibition of RSK2 activity was generated and IC_50_ values were determined. To confirm inhibition of RSK2 activity, we also repeated the kinase assay with a secondary RSK substrate, S6K.

### Cell culture

The triple-negative breast cancer cell lines SUM149 (Asterand, Ann Arbor, MI) and MDA-MB-231 (American Tissue Culture Collection, Manassus, VA) were grown as previously described [5]. Primary relapsed TNBC cells, x43, were a generous gift from Dr. John Hassell (McMaster University, Hamilton, ON) and were cultured in RPMI containing 10% (v/v) fetal bovine serum plus 100 units/ml penicillin, 100 units/ml streptomycin and 0.5 μg/ml fungizone amphotericin B. All experimentation involving human cells were done in accordance with the Helsinki guidelines and approved through McMaster University ethics committee.

### Immunofluorescence and western blotting

SUM149 cells were plated on 8-well multi-chamber slides (40,000 cells/well) and treated with 10 μM of each lead compound for 24 h. Immunofluorescence was conducted as previously described [17] using P-YB-1^S102^ and YB-1 antibodies (Cell Signaling, Danvers, MA) with Alexa-Fluor 488 (Invitrogen, Burlington, ON) secondary antibody. Images were acquired on an Olympus BX61 microscope and analyzed using ImageJ (NIH, Bethesda, MD). For western blotting, cell lysates were collected after 24-72 h drug treatments or 48-96 h siRNA treatment and immunoblotting was performed as described previously [10, 41] using RSK1; 1:1000 (Santa Cruz Biotechnology, Santa Cruz, CA), RSK2; 1:500 (Santa Cruz Biotechnology), YB-1; 1:2000 (Cell Signaling Technology, Boston, MA), YB-1; 1:1000 (Epitomics, Burlingame, CA), CD44; 1:1000 (Abcam, Cambridge, MA), Flag; 1:2000 (Sigma, Oakville, ON), P-YB-1^S102^; 1:1000 (Cell Signaling Technology), P-GSK3β^S9^; 1:1000 (Cell Signaling Technology), P-S6^S236^; 1:1000 (Cell Signaling Technology), Notch4, 1:500 (Santa Cruz Biotechnology), α/β-Tubulin; 1:1000 (Cell Signaling Technology), Vinculin; 1:1000 (Upstate, MA) and Pan-actin; 1:1000 (Cell Signaling Technology).

### Transfections

To investigate the effect of altering YB-1 on expression of Notch isoforms, SUM149 cells were transfected with three distinct siRNAs against YB-1 or scramble control (20 nM) for 96 h as described in [17]. SUM149 cells were transiently transfected with 4 μg of Flag:EV, Flag:YB-1-WT or Flag:YB-1-D102 plasmids and subsequently harvested after 96 h [17]. MDA-MB-231 and x43 cells were treated with (20 nM) siRSK1 or siRSK2, (Qiagen, Mississauga, ON) or siYB-1#1 or siYB-1#3 or control scramble (Darmacon, Chicago, Illinios) for 72 h. Both RSK1 and RSK2 siRNA’s have been compared to alternate sequences targeting each isoform and found to have comparable knockdown and phenotypic effects [10].

### Real-time quantitative reverse transcription PCR

RNA was isolated using RNeasy mini kit (Qiagen, Mississagua, ON). SUM149 cells were treated with DMSO or 10 μM of kaempferol, apigenin or luteolin for 48 h. BI-D1870 (10 μM) was used as a control. The RNA was reverse transcribed and amplified using CD44 specific primers and probes (Applied Biosystems, Foster City, CA) as previously described [17]. Ribosomal mRNA was quantified as a housekeeping gene (Applied Biosystems). Taqman Gene Expression Assays designed for Notch1, Notch2, Notch3 and Notch4 specific primers and probes (Applied Biosystems, Foster City, CA) were used with PPIA (Applied Biosystems, Foster City, CA) as the internal control.

### Monolayer, mammosphere and soft agar growth assays

Monolayer growth assays were performed with 5,000 (SUM149) or 3,000 (MDA-MB-231) cells/well/96 well plate. Cells were treated with DMSO, 10 or 100 μM of the drugs and counted by high-content screening as previously described [65] after 72 h. For combination monolayer drug treatments; 5,000 SUM149 cells/well/96 well plate were seeded and treated at 24 h with various combinations of luteolin (0, 10 or 25 μM) and paclitaxel (0,1, 5 or 10 nM). Cells were fixed at 72 h and stained for P-YB-1^S102^ (Cell Signaling, Danvers, MA) with Alexa-Fluor 488 (Invitrogen, Burlington, ON) secondary and CD44-PE conjugated (BD Pharmingen, Mississauga, ON) and signal was quantified using Cellomics ArrayScan VTI as previously described [10]. Soft agar assays were performed as previously described [66]. Compounds (10 μM) were added at seeding into the top layer and colonies were counted after 28-30 d. Percent change in growth was compared to DMSO control. Mammosphere assays were performed as previously described [17] in Mammocult media (Stemcell Technologies, Vancouver, BC). Additionally, serial passaging of mammospheres (with fresh kaempferol added with each passage) and daily repeated dosing was conducted with 10 μM kaempferol and spheres were counted after 7 d. Growth and mammosphere assays were performed as described above for primary x43 cells. Cells were seeded at 5,000 cells/well/96 well plate and analyzed at 72 h for monolayer growth. For mammosphere assays x43 cells were seeded at 20,000 cells/well and treated with DMSO, 10 or 50 μM or BI-D1870 (10 μM) as a positive control.

### FACS sorting for CD44+/CD24- SUM149 cells

SUM149 cells were sorted for the top 10% CD44^+^/CD24^−^ TICs as previously described [17] using anti-CD44 conjugated to PE (BD Biosciences, Mississauga, ON) and anti-CD24 conjugated to FITC (Stemcell Technologies, Vancouver, BC). Immunofluorescence of P-YB-1^S102^ as well as monolayer and mammosphere assays were performed using the CD44^+^/CD24^−^ TIC-enriched population as described above.

### Apoptosis assays

SUM149 and x43 cells (5,000 cells/well/96 well plate) were treated with DMSO, 10 or 50 μM luteolin BI-D1870 (10 μM) or combined with paclitaxel (10 nM) for 72 h. PI-uptake was quantified using the Cellomics ArrayScan VTI as described in [10].

### NanoString gene expression profiling

RNA (100-250 ng) from breast cancer cell lines was analyzed using the nCounter Gene Expression Analysis system at the Centre for Translational and Applied Genomics (CTAG) at the BC Cancer Agency (Vancouver, BC). A custom CodeSet containing probes for ER (RefSeq NM_000125.2), PR (RefSeq NM_000926.4), HER2 (RefSeq NM_004448.2), EGFR (RefSeq NM_005228.3), KRT5 (RefSeq NM_000424.2) and KRT6A (RefSeq NM_005554.3) was synthesized by NanoString Technologies (Seattle, WA, USA). All procedures related to mRNA quantification including sample preparation, hybridization, detection, scanning and data normalization were carried out as recommended by NanoString Technologies.

## Supplementary Figures and Tables




