# Supplementary material for: Luteolin is a novel p90 ribosomal S6 kinase (RSK) inhibitor that suppresses Notch4 signaling by blocking the activation of Y-box binding protein-1 (YB-1)
Source: Oncotarget. 2013 Feb 27;4(2):329–45. doi: 10.18632/oncotarget.834 (PMC3712578; doi:10.18632/oncotarget.834)
Supplement: Supplementary file 1 [file oncotarget-04-329-s001.pdf]

Luteolin is a novel p90 ribosomal S6 kinase (RSK) inhibitor that suppresses Notch4 signaling by blocking the activation of Y-box binding protein-1 (YB-1) – Reipas et al

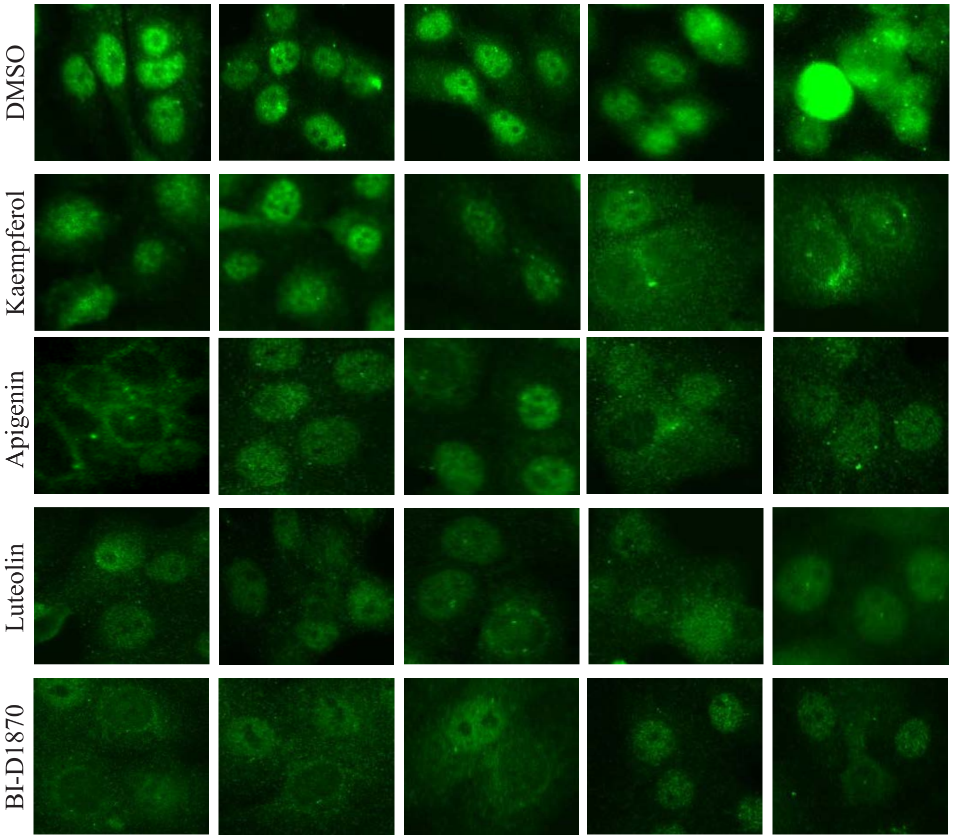

Supplemental Figure 1: Lead compounds block nuclear translocation of YB-1. Additional immunofluorescence images of SUM149 cells treated with 10 µM of each lead compound for 24 h and stained for P-YB-1^{S102}.

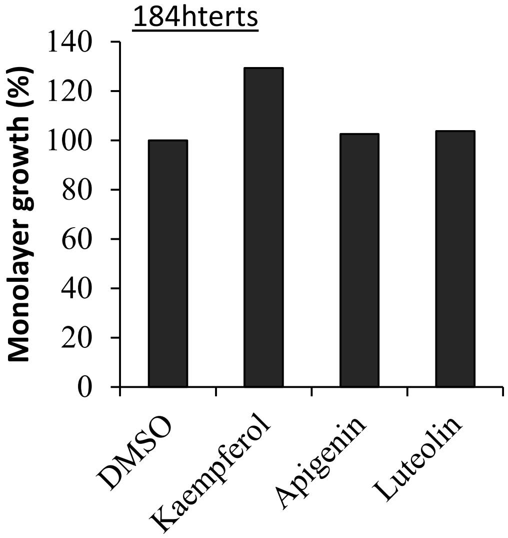

Supplemental Figure 2: The effect of lead compounds on normal immortalized epithelial cells (184hterts). Growth of 184htert cells was not inhibited by 10 µM of drug candidates at 72 h.

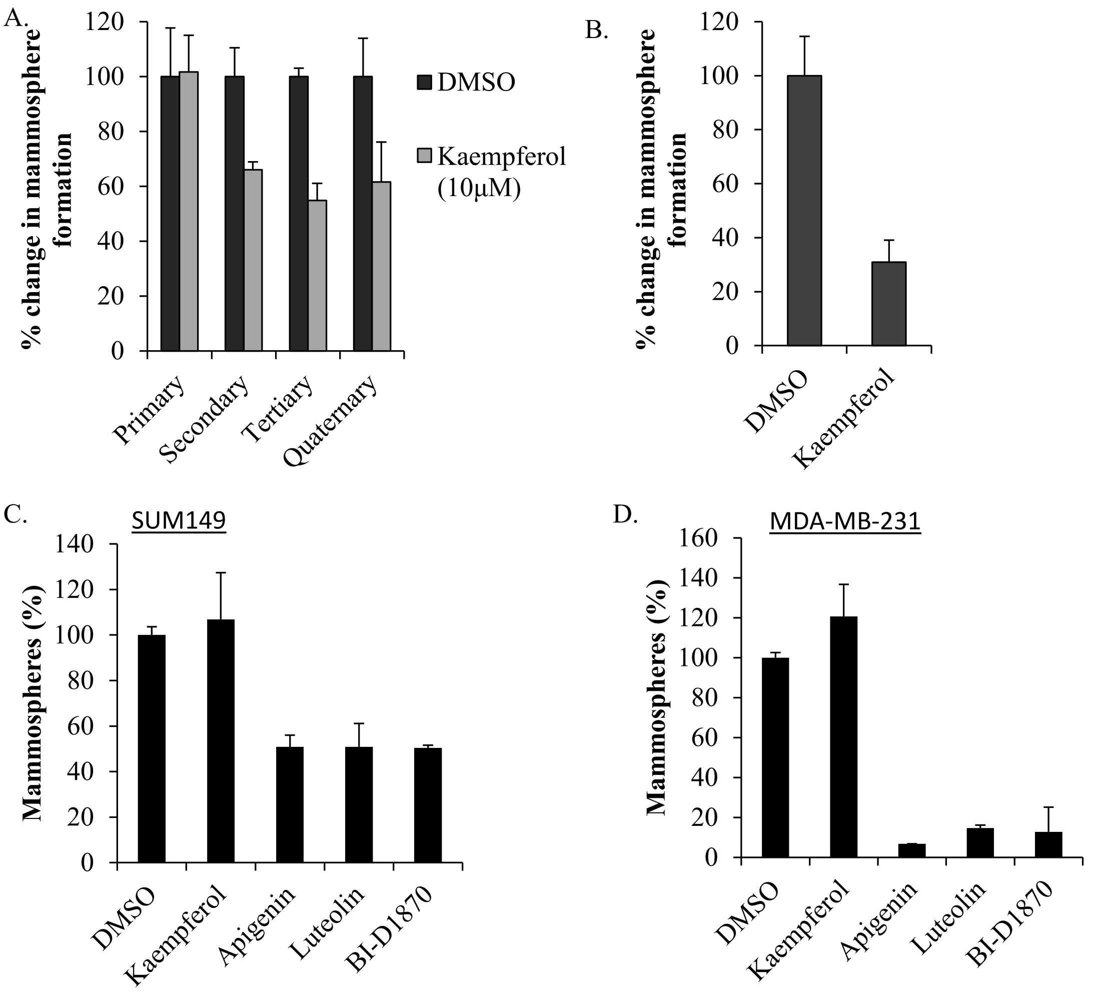

Supplemental Figure 3: The effect of lead compounds on mammosphere formation. A) The effect of kaempferol (10 µM) on serially passaged mammospheres. SUM149 cells were passaged every 7 days in fresh mammocult media containing kaempferol (10 µM). The percent change was calculated relative to primary DMSO control. B) The effect of kaempferol on SUM149 mammosphere formation when added daily (10 µM for 7 d). C) The effect of lead compounds on regression of formed mammospheres in the SUM149 and D) MDA-MB-231 cell lines. For this assay, mammospheres were grown for 3 d prior to drug treatment (10 µM) and were counted after a further 4 d.

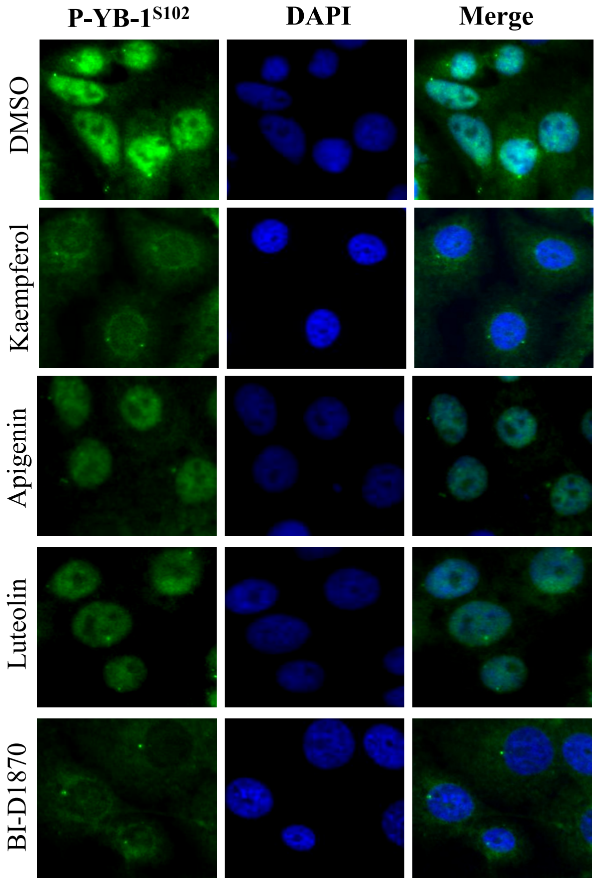

Supplemental Figure 4: Lead compounds block nuclear translocation of YB-1 in TIC-enriched populations. Immunofluorescence images of $CD44^+/CD24^-$-sorted SUM149 cells treated with 10 µM of each compound for 24 h and subsequently stained for P-YB-1^{S102} and DAPI.

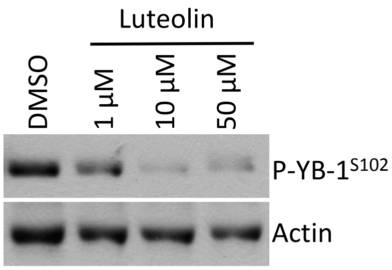

Supplemental Figure 6: Luteolin decreases P-YB-1S102 in a dose dependent manner. P-YB-1^{S102} decreases in a dose-dependant manner upon treatment with luteolin for 72 h in SUM149 cells.

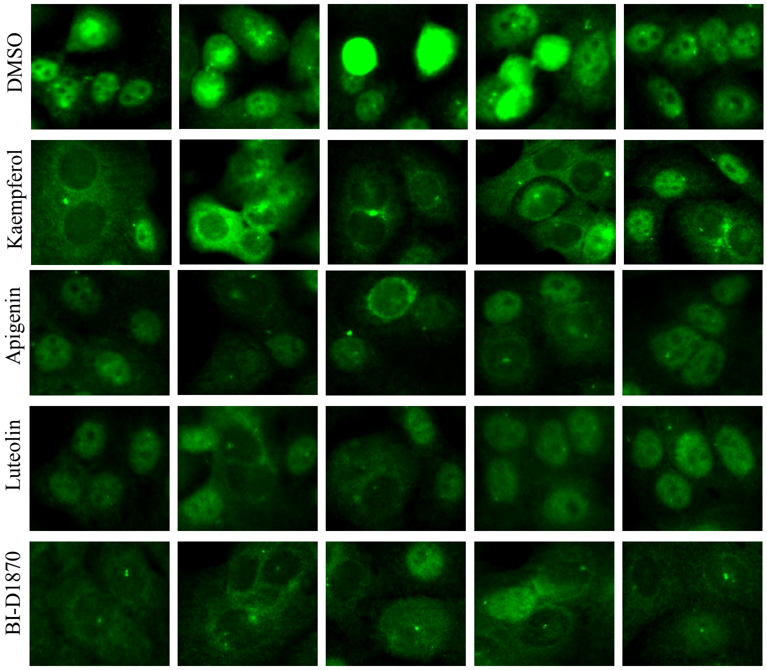

Supplemental Figure 5: Lead compounds block nuclear translocation of YB-1 in TIC-enriched populations: Additional immunofluorescence images. $CD44^+/CD24^-$-sorted SUM149 cells treated with 10 µM of each compound for 24 h and subsequently stained for P-YB-1^{S102}.

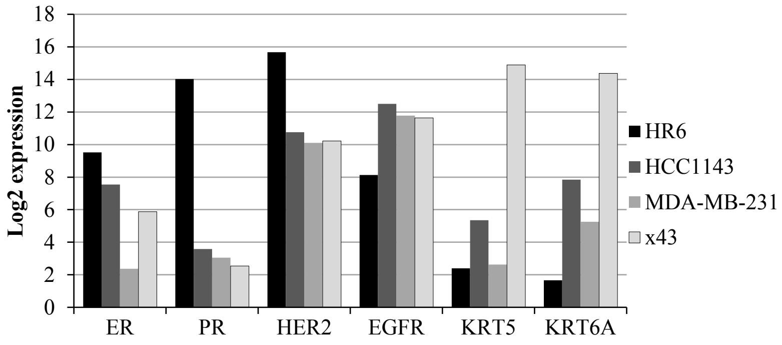

Supplemental Figure 7: Classification of x43 primary human TNBC. Cell surface marker expression of x43 cells was compared to Her-2 over-expressing (HR6) and TNBC (HCC1143 and MDA-MB-231) cell lines by NanoString technology.

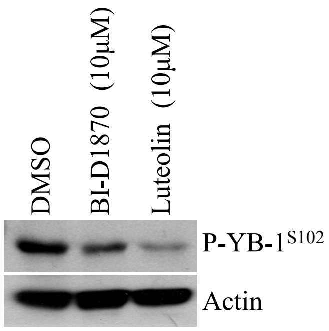

Supplemental Figure 8: Luteolin and BI-D1870 decrease P-YB-1^{S102} in x43 cells. Treatment with luteolin (10 µM) or BI-D1870 (10 µM) reduced P-YB-1^{S102} levels. Cell lysates were collected after 24 h drug treatment

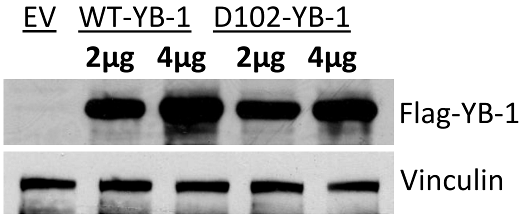

Supplemental Figure 9: Control blot validating Flag transgene expression. SUM149 cells transfected with EV, Flag:YB-1^{WT} or Flag:YB-1^{D102} demonstrated expression of the transgene at 96 h.

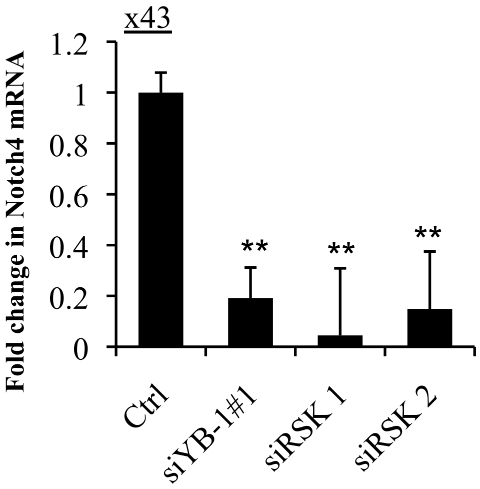

Supplemental Figure 10: Knockdown of YB-1, RSK1 or RSK2 reduces Notch4 mRNA in x43 cells. Primary x43 cells were treated with siYB-1, siRSK1 or siRSK2 for 96 h. Notch4 mRNA expression was quantified using qRT-PCR.
