# Supplementary material for: Luteolin is a novel p90 ribosomal S6 kinase (RSK) inhibitor that suppresses Notch4 signaling by blocking the activation of Y-box binding protein-1 (YB-1)
Source: Oncotarget. 2013 Feb 27;4(2):329–45. doi: 10.18632/oncotarget.834 (PMC3712578; doi:10.18632/oncotarget.834)
Supplement: Supplementary file 2 [file oncotarget-04-329-s002.pdf]

Luteolin is a novel p90 ribosomal S6 kinase (RSK) inhibitor that suppresses Notch4 signaling by blocking the activation of Y-box binding protein-1 (YB-1) – Reipas et al

Supplemental Table 1: Results of primary screens determined compounds that had significant inhibition (>20%) of RSK1 kinase activity. Shaded agents were also identified through ICM molecular docking of the Prestwick compounds to a homology model of RSK1 and were selected for validation in subsequent assays.

Compound	Inhibition of RSK1 (%) against the YB-1 peptide as the substrate
Kaempferol	52
Phenindione	48
Ellipticine	40
Myricetin	39
Estriol	36
Nifuroxazide	36
Menadione	35
Piracetam	33
Kinetin	31
Pyrvinium pamoate	29
Methoxy-8-psoralen	27
Oxantel pamoate	27
Progesterone	27
α-Solanine	26
Dinoprost trometamol	25
Estradiol-17 beta	24
Hesperidin	24
Amrinone	23
Felodipine	23
Hydroquinine hydrobromide hydrate	23
Isocarboxazid	23
Riboflavine	23
Primaquine diphosphate	22
Pyrimethamine	22
Apigenin *	21
Cyclacillin	21
Luteolin *	21
Prochlorperazine dimaleate	21
Sulfinpyrazone	21
Thiorphan	21
Sulfaphenazole	20
Todralazine hydrochloride	20

- also identified in molecular docking

Supplemental Table 2: Molecular docking supports the ability of drugs to block RSK1 in additional crystal structures. Binding models for the lead compounds in relationship to the RSK1 NTKD co-crystallized to staurosporine, and puravalnol A. The Glidescore and rank of the lead compounds in the Prestwick Library are also shown.

	Docking using RSK1 N-terminal domain conformation co-crystallized with:					
	Staurosporine (2ZRQ.pdb)			Purvalanol A (2Z7S.pdb)		
	sp	rank[1]	%[2]	sp	rank	%
Kaempferol	-8.17	6	0.50	-7.03	90	7.50
Apigenin	-8.14	7	0.58	-8.38	4	0.33
Luteolin	-7.84	30	2.50	-7.45	39	3.25

[1] the ranking of the compound among the 1120 Prestwick Chemical Library
[2] the percentage of the compound among the 1120 Prestwick Chemical Library

Supplemental Table 3: Kaempferol, apigenin and luteolin block RSK1 and RSK2 kinase activity. Lead candidates were screened in the A) RSK1 and the B) RSK2 kinase assay using an alternate RSK substrate, S6K.

A

	**Inhibition of RSK1 (%) against the S6K peptide as the substrate**						
**Compound**	**0.001μM**	**0.01μM**	**0.1μM**	**1.0μM**	**10μM**	**100μM**	**IC50**
Kaempferol	1	3	15	43	79	92	1.48μM
Apigenin	0	5	15	38	65	74	3.78μM
Luteolin	1	8	17	38	72	83	2.25μM
BI-D1870	16	35	61	84	94	99	0.03μM

B

	**Inhibition of RSK2 (%) against the S6K peptide as the substrate**						
**Compound**	**0.001µM**	**0.01µM**	**0.1µM**	**1.0µM**	**10µM**	**100µM**	**IC50**
Kaempferol	3	9	22	51	86	96	0.76µM
Apigenin	-4	3	6	32	70	87	3.48µM
Luteolin	3	5	8	20	66	87	4.71µM
BI-D1870	10	30	63	92	100	100	0.03µM

Supplemental Table 4: Predicted binding of luteolin to 252 known drug targets. 3D structures from PDB were used to dock luteolin using Glide and ICM. Targets were ranked based on pfm filters and ICM score.

**Predicted binding of luteolin to 252 known drug targets**			
**Protein**	**Score**	**pmfscore**	**drug rank**
**RSK** ***p90 ribosomal S6 kinase***	**-29**	**-130**	**1**
**DAPK1** *DAPK1 death-associated protein kinase 1*	-36.6	-121.9	2
**SYK** Spleen tyrosine kinase	-36.7	-121.7	7
**PNMT** *phenylethanolamine N-methyltransferase*	-37.9	-112.9	12
**NR3C2** *mineralocorticoid receptor*	-42.5	-126.9	17
**KIT** c-kit receptor	-33.3	-136.8	31
